# Low Circulating TRAIL Levels Are Associated with Increase of Resistin and Lipocalin-2/ngal Adipokines in Postmenopausal Women

**DOI:** 10.1155/2017/5356020

**Published:** 2017-09-05

**Authors:** Veronica Tisato, Paola Secchiero, Gloria Bonaccorsi, Carlo Bergamini, Pantaleo Greco, Giorgio Zauli, Carlo Cervellati

**Affiliations:** ^1^Department of Morphology, Surgery and Experimental Medicine and LTTA Centre, University of Ferrara, Via Fossato di Mortara 70, 44121 Ferrara, Italy; ^2^Department of Morphology, Surgery and Experimental Medicine, Section of Obstetrics and Gynecology, University of Ferrara, Via Aldo Moro 8, 44124 Ferrara, Italy; ^3^Department of Biomedical and Specialist Surgical Sciences, Section of Medical Biochemistry, Molecular Biology and Genetics, University of Ferrara, Via Borsari 46, 44121 Ferrara, Italy

## Abstract

**Objective:**

Tumor necrosis factor- (TNF-) related apoptosis-inducing ligand (TRAIL) is attracting attention for its role in the physiopathology of metabolic disease/diabetes. Evidence suggests that it might protect against metabolic abnormalities driven by obesity-induced dysregulated secretion of adipokines, but this role of TRAIL has not yet been fully established. On this basis, we aimed to investigate the potential association between TRAIL and adipokine levels in a cohort of subjects in which age/gender/hormonal interferences were excluded.

**Methods:**

Serum levels of TRAIL and a panel of adipokines were measured in postmenopausal women (*n* = 147) stratified according to waist circumference measures as normal, overweight, or obese. The panel of adipokines included interleukin- (IL-) 6, IL-8, IL-1*β*, adipsin, lipocalin-2/neutrophil gelatinase-associated lipocalin (ngal), TNF-alpha, monocyte chemoattractant protein-1, plasminogen activator inhibitor-1, hepatocyte growth factor, resistin, leptin, adiponectin, and nerve growth factor.

**Results:**

Low serum TRAIL concentration (deciles I–IV) was significantly and inversely correlated with resistin and lipocalin 2/ngal levels (*r* = −0.502 and *p* < 0.001 and *r* = −0.360 and *p* < 0.01, resp.). Both associations retained their statistical significance after adjustment for confounding factors, such as waist circumference and age.

**Conclusions:**

Our data indicate a link between low circulating levels of TRAIL and markers of obesity-induced diseases (resistin and lipocalin-2/ngal), highlighting a new potential axis of TRAIL functions.

## 1. Introduction

Tumor necrosis factor- (TNF-) related apoptosis-inducing ligand (TRAIL) is a member of the TNF superfamily expressed by various cell types, mostly in the immune system [1]. There have been many studies focused on the role of TRAIL in cancer because one of the best-characterized properties of this molecule is its ability to induce apoptosis in tumors and immortalized cells [1]. However, the action of TRAIL is not exclusively exerted on transformed cells, and it may be effective in primary cells, including immune cells [2, 3]. Of note, the activation of TRAIL receptors may activate nonapoptotic and prosurvival/proliferation signals such as those mediated by the mitogen-activated protein kinases, PI3K/Akt and ERK pathways, and transcription factor nuclear factor-*κ*B, which are pathways involved in the regulation of developmental and inflammatory processes [4]. Recent findings have highlighted a potential role of this molecule in a number of diseases other than cancer, such as diabetes, atherosclerosis, and hypertension [5, 6].

The accumulation of subcutaneous and visceral fat in abdominal depots is accompanied by marked alterations in the secretory profile of adipose tissue [7, 8]. A heterogeneous collection of molecules, including inflammatory cytokines/chemokines, hormone-like molecules, and other bioactive compounds overall defined as adipokines, is secreted by fat and nonfat cells in adipose tissue in obese individuals. The resulting shift from an anti-inflammatory (lean) to a proinflammatory (obese) phenotype is coupled with a significant local and systemic increase in some of these key cytokines, including interleukin 6 (IL-6), IL-1, IL-8, and tumor necrosis factor *α* (TNF-*α*), which critically contribute to insulin resistance development [9]. In addition to these inflammatory molecules, several adipokines such as lipocalin-2/neutrophil gelatinase-associated lipocalin (ngal), adiponectin, resistin are secreted and are known to be responsible, although at different extents and directions, for the low level of systemic inflammation present in metabolic syndrome associated with obesity. In this context, TRAIL exerts several effects on the homeostasis of adipose tissue such as a regulatory action on adipocyte metabolism and the inhibition of adipogenic differentiation, as demonstrated in human preadipocytes with the downregulation of adipogenic transcription factors and decreased lipid accumulation [10]. However, although TRAIL and adipokines are both involved in the pathogenesis of metabolic alteration, obesity, and diabetes, no potential associations between them have been reported to date. Many lines of preclinical evidence have indeed highlighted that TRAIL might act by downregulating the low-grade chronic inflammation associated with the onset and progression of (central) obesity-related diseases, *in primis* type II diabetes mellitus (T2DM) [2, 11–13]. Nonetheless, a thorough understanding of the physiopathological potential effects of TRAIL on metabolic and inflammatory dysregulation associated with fat accumulation from obesity up to T2DM is still lacking. A first step in this direction has been achieved by recent studies performed on different population cohorts, showing that the circulating levels of TRAIL are positively and independently associated with overall adiposity [14–18]. In this line, the present study aims to shed light on the mechanisms linking TRAIL to obesity and obesity-induced abnormalities/diseases by exploring the possible associations between TRAIL and a panel of selected adipokines. Of note, because our previous findings clearly showed that circulating TRAIL levels are affected by age, gender, and hormonal status [19], we intentionally performed this study using a cohort of postmenopausal women among whom these features were consistent and who were stratified according to waist circumference measures as normal, overweight, or obese.

## 2. Materials and Methods

### 2.1. Study Subjects

Between 2008 and 2016, a cohort of women was randomly enrolled among outpatients undergoing bone densitometry testing at the Menopause and Osteoporosis Centre of the University of Ferrara (Ferrara, Italy). The main aim of the research protocol was to explore the relationship between menopause-related changes in body fat composition and the pattern of inflammatory and oxidative mediators that are potentially involved in typical postmenopausal clinical complications. The protocol conformed to the Code of Ethics of the World Medical Association (Declaration of Helsinki) and was performed according to the guidelines for Good Clinical Practice (European Medicines Agency). The study was approved the local Ethics Committee (S. Anna University Hospital of Ferrara), and written informed consent was obtained from each patient during the first visit at baseline for possible inclusion in the study. No personal information was available to the authors of the study in order to protect the anonymity of the patients. Clinical and laboratory data were collected from each patient at admission, including a complete medical history and physical examination. Each participant underwent measurement of anthropometric parameters such as weight, standing height (for the calculation of body mass index, BMI), and waist circumference (WC) by trained personnel. In particular, WC was measured at the midpoint between the lower margin and the last rib and the top of the iliac crest on standing subjects at the end of gentle expiration by a single technician.

From the initial sample of 513 subjects consisting of reproductive age/perimenopause/postmenopausal women aged 20–72 years (details on the selection criteria are in [20]), 147 women were selected for the present study. Women were included if at the time of recruitment they were in a postmenopausal status defined as the cessation of menses for at least 1 year in accordance with the recent ReSTAGE's modification of the Stages of Reproductive Aging Workshop (STRAW) staging criteria [20, 21]; not using supplements containing antioxidants such as vitamins E and C or following a vegan diet; not affected by pathological conditions such as cancer, malabsorption, diabetes type I and II, and cardiovascular diseases (i.e., history of stroke and/or heart attack, previous diagnosis of cardiovascular diseases according to latest WHO classification http://www.who.int/mediacentre/en/); not under pharmacological treatment such as with thyroid hormones, diuretics, weight loss agents/drugs, statins, insulin, and antiglycemic and antiosteoporotic drugs (e.g., denosumab) during the last month prior to blood sampling; and not undergoing hormone therapy.

Fresh peripheral blood samples were collected by venipuncture into Vacutainer tubes without anticoagulant after an overnight fast. After 30 min of incubation at room temperature, the blood samples were centrifuged at 4.650 ×g for 20 min, and sera were collected and stored in single-use aliquots at −80°C until analysis.

### 2.2. Biochemical Assays

Serum samples were frozen and thawed only once before performing the MILLIPLEX MAP Human Adipokine Panels HADK1MAG-61K and HADK2MAG-61K (Merck Millipore, Billerica, MA) that allow the simultaneous quantification of the following human adipokines: resistin (intra-assay coefficient of variability [CV] = 3%; interassay CV = 14%); lipocalin-2/neutrophil gelatinase-associated lipocalin (NGAL) (intra-assay CV = 4%; interassay CV = 12%); total plasminogen activator inhibitor-1 (PAI-1 total) (intra-assay CV = 5%; interassay CV = 14%); adipsin (intra-assay CV = 2%; interassay CV = 6%); nerve growth factor (NGF) (intra-assay CV = 4%; interassay CV = 11%); adiponectin (intra-assay CV = 4%; interassay CV = 10%); IL-8 (intra-assay CV = 3%; interassay CV = 14%); TNF-*α* (intra-assay CV = 3%; interassay CV = 16%); IL-6 (intra-assay CV = 2%; interassay CV = 10%); monocyte chemotactic protein 1 (MCP-1) (intra-assay CV = 2%; interassay CV = 11%); IL-1*β* (intra-assay CV = 7%; interassay CV = 12%); hepatocyte growth factor (HGF) (intra-assay CV = 3%; interassay CV = 11%); and leptin (intra-assay CV = 5%; interassay CV = 13%).

Samples were processed in duplicate following the manufacturer recommended protocols and read on a MAGPIX instrument equipped with the MILLIPLEX-Analyst Software using a five-parameter nonlinear regression formula to compute sample concentrations from the standard curves. Quality controls provided in the multiplex kits were used to validate the assay performance. Within the adipokines assayed, IL-1*β* was the only one undetectable in more than the 80% of samples and therefore this cytokine was not considered in the following statistical analyses.

Serum TRAIL levels were analyzed in duplicate by using a specific ELISA kit (R&D Systems, Minneapolis, MN) in agreement with the manufacturer's instructions as previously described [22].

### 2.3. Statistical Analysis

Continuous variables were first analyzed for the normal distribution by the Shapiro-Wilkinson test. Comparisons between groups were performed using one-way analysis of variance (ANOVA) (Tukey post hoc test) and Kruskal-Wallis (Wilcoxon–Mann–Whitney test followed by Bonferroni adjustment) for normally and nonnormally (if the skewed distribution persisted after base-10 logarithm transformation) distributed variables, respectively. Simple correlation analyses were performed using Pearson's and Spearman's tests for normally and nonnormally distributed variables, respectively. Since the distribution of some variables of interest became normal upon base-10 logarithm transformation, we used the log values for correlation analyses. Multiple regression analysis was performed to determine whether the found associations were independent of potential confounding factors. Preliminary multiple regression analyses were performed to assess multicollinearity among variables included in the multivariate analyses. Values of variance inflation factor (VIF) above 2.5 were considered indicative of the presence of this statistical problem. Two-tailed probability values < 0.05 were considered statistically significant.

## 3. Results

The main anthropometric and laboratory characteristics of the postmenopausal cohort of subjects enrolled in the present study are summarized in Table 1. In order to investigate the relationship between the serum levels of TRAIL and adipokines, waist circumference was evaluated for correlation studies, and the cohort was stratified according to abdominal obesity (WHO classification) in normal weight (waist circumference ≤ 79.9 cm), overweight (80–87.9 cm), and obese (≥88 cm) groups, which were equally represented (Table 1).

In line with the current literature [23], most of the cytokines analyzed showed a trend toward increased levels across the different groups of subsets considered (Table 2). Regardless of the overall ANOVA outcome, the post hoc analysis revealed a significant (*p* < 0.05) increase in TRAIL in overweight (>88 cm) compared to normal (≤79.9 cm) women, and Pearson's test highlighted a significant (*r* = 0.227, *p* = 0.02) correlation between TRAIL and waist circumference, in line with a previous study performed in a different cohort of subjects [15] (Table 2).

**Figure 1:**
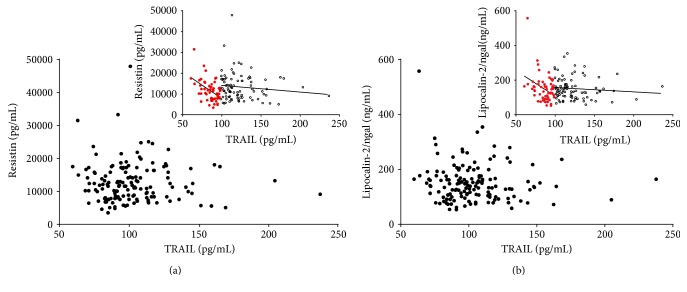
Correlation between TRAIL and resistin and between TRAIL and lipocalin-2/ngal in the whole sample (*n* = 147). Scatter plot showing the relationship between the circulating levels of TRAIL and resistin (a) and between circulating levels of TRAIL and lipocalin-2/ngal (b) in the whole cohort of samples. Insets show the two different relationship between TRAIL and resistin (inset in (a)) and between TRAIL and lipocalin-2/ngal (inset in (b)) after division of the whole population in two groups according to the upper limit of TRAIL decile IV (TRAIL: 98.59 pg/mL). Empty square data points = samples with TRAIL levels higher than TRAIL decile IV; full red circle data points = samples with TRAIL levels lower than TRAIL decile IV.

**Figure 2:**
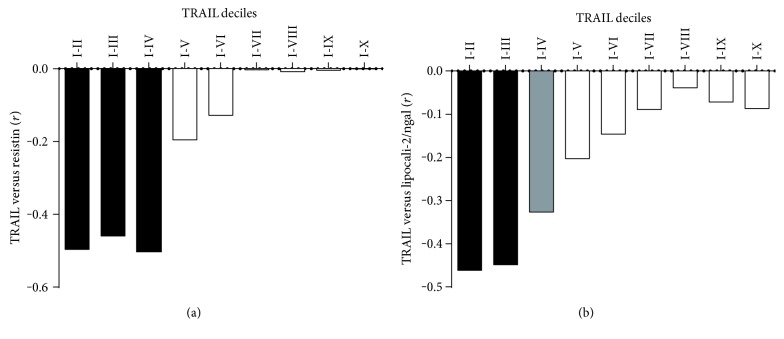
Pearson's correlation coefficient (*r*) values trend within cumulating TRAIL deciles. Box plot showing the correlation coefficients for the relationship TRAIL versus resistin (a) and TRAIL versus lipocalin-2/ngal (b) calculated dividing subjects in deciles based on TRAIL concentration.

To address the main aim of the present study, we then evaluated the possible correlations between TRAIL and adipokines (Supplementary Table 1 available online at https://doi.org/10.1155/2017/5356020). Despite the correlation results, the graphical representation of the distributions of the data generated by plotting adipokines as a function of TRAIL suggested another interpretation. In particular, the scatter plots linking TRAIL and resistin and linking TRAIL and lipocalin-2/ngal (Figures 1(a) and 1(b)) both showed two sharply separated trends. In detail, at relatively low levels of TRAIL, the data were clustered and formed a dense “cloud,” suggesting the existence of a significant correlation between the two variables. By contrast, at higher TRAIL levels, the data showed a clear scattered distribution, suggesting that the correlation had disappeared. To visualize and statistically evaluate this observation, we subdivided the sample according to deciles of TRAIL and analyzed the correlations in cumulating deciles (Figures 2(a) and 2(b)). By following this approach, we found that in the subset of subjects included in the first four deciles (TRAIL deciles I–IV, *n* = 58), TRAIL was strongly and inversely associated with resistin (*r* = −0.502, *p* < 0.001, *R*^2^ = 0.252) and, although less closely, with lipocalin-2/ngal (*r* = −0.360, *p* < 0.01, *R*^2^ = 0.138). The addition of a further decile led to a decrease in correlation strength and the loss of significance (*r* = −0.196 and *r* = −0.206 for TRAIL versus resistin and versus lipocalin-2/ngal, resp.). The further analysis performed by dividing the population into two subgroups defined as below and above than the upper limit of the IV decile of TRAIL (level: 98.59 pg/mL) revealed two different linear trends, which were evident both graphically (insets in Figures 1(a) and 1(b)) and geometrically as summarized below:

- TRAIL versus resistin—linear regression equations: deciles I–IV: *y* = −256 (±64) + 33,463 (±5577); deciles V–X: *y* = −31 (±29) + 17,556 (±3684)
- TRAIL versus lipocalin-2/ngal—linear regression equation: deciles I–IV: *y* = −2.49 (±1.063) + 402 (±109); deciles V–X: *y* = −0.257 (±0.322) + 185 (±39)

To verify the independence of these correlations, we performed two multiple regression analyses with age, hypertension, and waist circumference as covariates. We excluded BMI from the multiple regression analyses because of its collinearity with WC and its weaker correlation with the variables of interest. The statistical tests showed that resistin and lipocalin-2/ngal remained associated with TRAIL, with almost the same strength observed in the univariate analyses (*β* = −0.522 and *β* = −0.358, resp.), regardless of the considered potential interfering factors (Table 3).

## 4. Discussion

The role of TRAIL in weight gain-associated metabolic alterations and obesity has not been fully established, although the general view is that the cytokine might mediate adaptive biological responses to the obesity-induced perturbation of metabolism and immune homeostasis [2]. The rationale for this study arose from our recent finding of a positive association between circulating TRAIL and general (mostly central) fat mass in postmenopausal women [17], in line with the results of other studies performed in different cohorts of subjects [14–16]. We therefore aimed to explore a potential link between TRAIL and adipokines, which can be considered the key biochemical mediators of white adipose cells. To address this hypothesis, we evaluated the serum levels of a panel of adipokines that were selected because of their well-recognized function in low-grade inflammation and onset of metabolic disturbances, and we explored the possible associations with soluble TRAIL in a cohort of healthy postmenopausal women with different degrees of abdominal fat accumulation up to obesity. We found that resistin and lipocalin-2/ngal, two adipokines involved in obesity-associated dysmetabolic state and inflammation, were similarly and inversely associated with TRAIL when this cytokine was detected within the first IV deciles of circulating levels. Notably, these correlations were independent of differences in body fat mass (besides age and hypertension) or obesity categorization (women included in deciles I–IV were almost equally distributed, at *n* = 22 normal weight, *n* = 12 overweight, and *n* = 24 obese). That is, these findings suggest the presence of a “biological window” of TRAIL levels in which it is associated with the downregulation of the release of the two adipokines, regardless of the amount of adiposity present in the subjects. This explorative study revealed an association between TRAIL and adipokines in obesity-induced metabolic alterations, thus providing insight into new potential functions of TRAIL in this context.

Human resistin is almost exclusively produced by monocytes and macrophages, including those infiltrating adipose tissue. On the other hand, the cell sources of lipocalin-2/ngal range from hepatocytes, activated leukocytes, and several human epithelial tissues [24]. Both adipokines appear to promote insulin resistance together with local and systemic inflammation and to play a pathogenic role in CVD and T2DM. Of interest, experiments on transgenic animals have revealed that lipocalin-2-deficient mice show significantly decreased fasting glucose levels, decreased insulin levels, and improved insulin sensitivity [25]. Similarly, TRAIL deficiency leads to insulin resistance as the result of the significant impairment of *β*-cell function and marked islet macrophage infiltration in ApoE/TRAIL-knockout mice fed with a high-fat diet [13]. Along the same line but in a different preclinical model, exogenous TRAIL administration to mice fed with a high-fat diet was associated with a reduction in metabolic abnormalities and disease expression compared to TRAIL-free animals maintained under the same diet regimen [12]. In this context, the modulation of adipocyte metabolism and inhibition of differentiation seem to occur via inhibition of the peroxisome proliferator-activated receptor *γ* (PPAR*γ*) adipocyte transcription factor [10]. Moreover, it has been suggested that PPAR*γ* might play a key role in the physiological processes involving both resistin and lipocalin-2/ngal. On the one hand, lipocalin was found to be a selective modulator of PPAR*γ* activation [26], and on the other hand, the activation of this factor with a pharmacological agonist (e.g., thiazolidinediones) was found to affect the expression of several adipokines, including resistin [27]. Of interest, Zoller and colleagues have recently addressed the role of TRAIL in adipogenesis, showing that human recombinant TRAIL was able to interfere with adipogenesis differentiation by downregulating several key adipogenic transcription factors, including PPAR*γ*, C/EBP*α*, and C/EBP*δ*, highlighting an unknown function of TRAIL in the regulation of adipose tissue homeostasis [28].

The loss of the association between TRAIL and the two adipokines when TRAIL is at high levels might be the result of the onset of TRAIL resistance due to altered expression of TRAIL receptors/decoy receptors or dysfunctional TRAIL/TRAIL receptor signaling, but the full identification of the mechanisms responsible will require further investigations. In human studies, circulating TRAIL levels were found to be lower in newly diagnosed T2DM patients compared to those in healthy controls [29] and, by contrast, higher in those with more advanced and complicated disease [30]. Moreover, significantly increased TRAIL levels have been recently found in patients with vascular dementia [31], a cognitive disorder caused by T2DM and other cardio-metabolic pathological conditions [32]. Other clinical and epidemiological studies have suggested a protective prognostic role for high TRAIL levels in patients with acute myocardial infarction and advanced heart failure [33, 34]. On the other hand, we cannot exclude that the high levels of soluble TRAIL might also be an adaptive response in an attempt to balance central adipose-driven inflammation.

**Figure 3:**
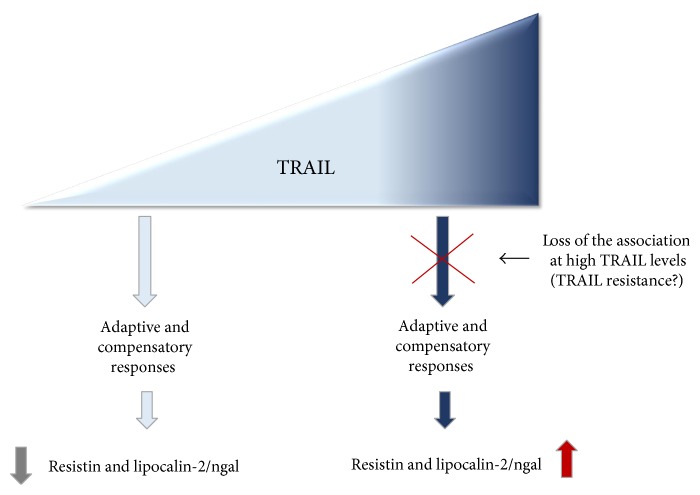
Schematic representation of the link between TRAIL and resistin/lipocalin-2 (ngal) in the context of obesity-induced metabolic alterations. The picture summarize the hypothesized axis between TRAIL and resistin and lipocalin-2/ngal leading to different outcomes according to TRAIL levels.

In conclusion, although we are aware of the potential limitations of this study (cross-sectional design), our results obtained in a cohort of subjects in which age/gender/hormonal interferences were excluded demonstrate a link between TRAIL and markers of obesity-induced alterations. However, further investigations are needed to determine whether low TRAIL level is a cause (as hypothesized in Figure 3) or a consequence of adipokine overexpression. We believe that a thorough characterization via a longitudinal study with the same subject sample as well as an evaluation of TRAIL and adipokines in different pathological contexts will provide new insights into TRAIL activity and its therapeutic potential.

## Supplementary Material

Supplementary Table 1. Correlation coefficient (r) between TRAIL and Adipokines.

## Figures and Tables

**Table 1:** Principal characteristics of the subjects.

Parameters	Value^a^
Number of subjects, *n*	147
Age, years	56 ± 4
Years since menopause, years	7 ± 5
Age at menopause, years	49 ± 5
Smoking, %	15 (10)
Hypertension, %	10 (7)
*Anthropometric features*	
BMI, kg/m^2^	25 ± 3
WC, cm	84 ± 10
Abdominal obesity category	
(i) Normal (WC ≤ 79.9 cm), *n*	47
(ii) Overweight (WC = 80–87.9 cm), *n*	43
(iii) Obese (WC ≥ 88 cm), *n*	57
*Sexual hormones*	
17*β*-estradiol (pg/mL)	11 (6–30)
FSH (mUI/mL)	71 ± 33

^a^Data are expressed as % within the group for categorical variables and number of subjects in brackets; mean ± standard deviation for continuous variables. BMI: body mass index; WC: waist circumference.

**Table 2:** Serum levels of adipokines and TRAIL according to abdominal obesity categories.

Parameters	Waist circumference (cm)			
	≤79.9 [*n* = 47]	80–87.9 [*n* = 43]	≥88 [*n* = 57]	*p* ^∗^
IL-8 (pg/mL)	2.7 ± 0.9	3.3 ± 0.9	3.4 ± 1.9	NS
TNF-*α* (pg/mL)	1.7 (1.3–2.4)	1.9 (1.2–2.6)	2.0 (1.4–2.7)	NS
IL-6 (pg/mL)	0.4 (0.3–0.8)	0.5 (0.3–0.7)	1.0 (0.4–1.7)^a,b^	<0.01
MCP-1 (pg/mL)	207 (147–306)	193 (158–276)	213 (166–278)	NS
Resistin (pg/mL)	11,051 ± 4628	13,398 ± 7721^a^	13,540 ± 5476^a^	<0.05
Adiponectin (*μ*g/mL)	80 (49–170)	69 (37–158)^a^	43 (22–57)^a,b^	<0.01
HGF (pg/mL)	215 (108–318)	306 (187–483)	377 (272–571)^a,b^	<0.01
NGF (pg/mL)	0.9 (0.7–1.2)	1.1 (0.9–1.5)	1.2 (0.8–1.6)	NS
Adipsin (*μ*g/mL)	3.7 (3.1–4.5)	3.9 (3.3–4.8)	4.6 (4.8–6.0)^a,b^	<0.01
Leptin (ng/mL)	6 (4–13)	14 (6–22)^a^	29 (13–39)^a,b^	<0.01
Lipocalin-2/ngal (ng/mL)	135 ± 48	157 ± 82	160 ± 60^a^	<0.05
PAI-1 total (ng/mL)	46 ± 22	53 ± 22	61 ± 22^a^	<0.01
TRAIL	100 ± 20	110 ± 23^a^	110 ± 25	NS

Data presented are expressed as mean ± standard deviation for normal continuous variables; median (interquartile range) for nonnormal continuous variables. ^∗^ANOVA (post hoc test: Tukey) or Kruskall-Wallis (post hoc test: Wilcoxon–Mann–Whitney test followed by Bonferroni correction). ^a^*p* < 0.05 versus normal; ^b^*p* < 0.05 versus overweight. IL: interleukin; PAI-1 total: plasminogen activator inhibitor-1; TNF-*α*: tumor necrosis factor alpha; MCP-1: monocyte chemoattractant protein; HGF: hepatocyte growth factor; ngal: neutrophil gelatinase-associated lipocalin; NGF: nerve growth factor.

**Table 3:** Ability of TRAIL to predict resistin or lipocalin-2/ngal changes within deciles I–IV evaluated by multiple regression analysis.

Regression model	Outcome variables	Explanatory variable	Unstandardized coefficient B	Standard error	Standardized coefficient *β*	Contribute to outcome variance
1	Resistin	TRAIL	−275.1	63.1	−0.522^∗∗^	0.252^#^
2	Lipocalin-2/ngal	TRAIL	−3.5	1.4	−0.360^∗^	0.138

Both multiple regression models include age, hypertension, and waist circumference. B = unstandardized regression coefficient; *β* = standardized regression coefficient. ^#^The squared semipartial correlation coefficient accounts for the proportion of variance in the dependent variable that is explained by the covariate. ^∗^*p* < 0.01; ^∗∗^*p* < 0.001.

## References

[1] Bernardi S., Secchiero P., Zauli G. (2012). State of art and recent developments of anti-cancer strategies based on TRAIL. *Recent Patents on Anti-Cancer Drug Discovery*.

[2] Bossi F., Bernardi S., Zauli G., Secchiero P., Fabris B. (2015). TRAIL modulates the immune system and protects against the development of diabetes. *Journal of Immunology Research*.

[3] Tisato V., Gonelli A., Voltan R., Secchiero P., Zauli G. (2016). Clinical perspectives of TRAIL: insights into central nervous system disorders. *Cellular and Molecular Life Sciences : CMLS*.

[4] Voltan R., Secchiero P., Casciano F., Milani D., Zauli G., Tisato V. (2016). Redox signaling and oxidative stress: cross talk with TNF-related apoptosis inducing ligand activity. *The International Journal of Biochemistry & Cell Biology*.

[5] Cheng W., Zhao Y., Wang S., Jiang F. (2014). Tumor necrosis factor-related apoptosis-inducing ligand in vascular inflammation and atherosclerosis: a protector or culprit?. *Vascular Pharmacology*.

[6] Bernardi S., Bossi F., Toffoli B., Fabris B. (2016). Roles and clinical applications of OPG and TRAIL as biomarkers in cardiovascular disease. *BioMed Research International*.

[7] Cervellati C., Pansini F. S., Bonaccorsi G. (2009). Body mass index is a major determinant of abdominal fat accumulation in pre-, peri- and post-menopausal women. *Gynecological Endocrinology: The Official Journal of the International Society of Gynecological Endocrinology*.

[8] Balistreri C. R., Caruso C., Candore G. (2010). The role of adipose tissue and adipokines in obesity-related inflammatory diseases. *Mediators of Inflammation*.

[9] Kwon H., Pessin J. E. (2013). Adipokines mediate inflammation and insulin resistance. *Frontiers in Endocrinology*.

[10] Keuper M., Wernstedt Asterholm I., Scherer P. E. (2013). TRAIL (TNF-related apoptosis-inducing ligand) regulates adipocyte metabolism by caspase-mediated cleavage of PPARgamma. *Cell Death & Disease*.

[11] Bartolo B. A. D., Chan J., Bennett M. R. (2011). TNF-related apoptosis-inducing ligand (TRAIL) protects against diabetes and atherosclerosis in Apoe^−/−^ mice. *Diabetologia*.

[12] Bernardi S., Zauli G., Tikellis C. (2012). TNF-related apoptosis-inducing ligand significantly attenuates metabolic abnormalities in high-fat-fed mice reducing adiposity and systemic inflammation. *Clinical Science*.

[13] Harith H. H., Morris M. J., Kavurma M. M. (2013). On the TRAIL of obesity and diabetes. *Trends in Endocrinology and Metabolism: TEM*.

[14] Choi J. W., Song J. S., Pai S. H. (2004). Associations of serum TRAIL concentrations, anthropometric variables, and serum lipid parameters in healthy adults. *Annals of Clinical and Laboratory Science*.

[15] Ashley D. T., O'Sullivan E. P., Davenport C. (2011). Similar to adiponectin, serum levels of osteoprotegerin are associated with obesity in healthy subjects. *Metabolism: Clinical and Experimental*.

[16] Biolo G., Secchiero P., Giorgi S. D., Tisato V., Zauli G. (2012). The energy balance positively regulates the levels of circulating TNF-related apoptosis inducing ligand in humans. *Clinical Nutrition*.

[17] Brombo G., Volpato S., Secchiero P. (2013). Association of soluble tumor necrosis factor-related apoptosis-inducing ligand (TRAIL) with central adiposity and low-density lipoprotein cholesterol. *PLoS One*.

[18] Cervellati C., Secchiero P., Bonaccorsi G., Celeghini C., Zauli G. (2014). Association of serum tumor necrosis factor-related apoptosis inducing ligand with body fat distribution as assessed by dual X-rays absorptiometry. *Mediators of Inflammation*.

[19] Zauli G., Tisato V., Melloni E. (2014). Inverse correlation between circulating levels of TNF-related apoptosis-inducing ligand and 17beta-estradiol. *The Journal of Clinical Endocrinology and Metabolism*.

[20] Hale G. E., Zhao X., Hughes C. L., Burger H. G., Robertson D. M., Fraser I. S. (2007). Endocrine features of menstrual cycles in middle and late reproductive age and the menopausal transition classified according to the staging of reproductive aging workshop (STRAW) staging system. *The Journal of Clinical Endocrinology and Metabolism*.

[21] Cervellati C., Pansini F. S., Bonaccorsi G. (2011). 17beta-estradiol levels and oxidative balance in a population of pre-, peri-, and post-menopausal women. *Gynecological Endocrinology: The Official Journal of the International Society of Gynecological Endocrinology*.

[22] Tornese G., Iafusco D., Monasta L. (2014). The levels of circulating TRAIL at the onset of type 1 diabetes are markedly decreased in patients with ketoacidosis and with the highest insulin requirement. *Acta Diabetologica*.

[23] Choe S. S., Huh J. Y., Hwang I. J., Kim J. I., Kim J. B. (2016). Adipose tissue remodeling: its role in energy metabolism and metabolic disorders. *Frontiers in Endocrinology*.

[24] Wu G., Li H., Zhou M. (2014). Mechanism and clinical evidence of lipocalin-2 and adipocyte fatty acid-binding protein linking obesity and atherosclerosis. *Diabetes/Metabolism Research and Reviews*.

[25] Law I. K., Xu A., Lam K. S. (2010). Lipocalin-2 deficiency attenuates insulin resistance associated with aging and obesity. *Diabetes*.

[26] Pereira V. H., Marques F., Lages V. (2016). Glucose intolerance after chronic stress is related with downregulated PPAR-gamma in adipose tissue. *Cardiovascular Diabetology*.

[27] Sharma A. M., Staels B. (2007). Review: peroxisome proliferator-activated receptor gamma and adipose tissue—understanding obesity-related changes in regulation of lipid and glucose metabolism. *The Journal of Clinical Endocrinology and Metabolism*.

[28] Zoller V., Funcke J. B., Keuper M. (2016). TRAIL (TNF-related apoptosis-inducing ligand) inhibits human adipocyte differentiation via caspase-mediated downregulation of adipogenic transcription factors. *Cell Death & Disease*.

[29] Bisgin A., Yalcin A. D., Gorczynski R. M. (2012). Circulating soluble tumor necrosis factor related apoptosis inducing-ligand (TRAIL) is decreased in type-2 newly diagnosed, non-drug using diabetic patients. *Diabetes Research and Clinical Practice*.

[30] Chang Y. H., Lin K. D., He S. R., Hsieh M. C., Hsiao J. Y., Shin S. J. (2011). Serum osteoprotegerin and tumor necrosis factor related apoptosis inducing-ligand (TRAIL) are elevated in type 2 diabetic patients with albuminuria and serum osteoprotegerin is independently associated with the severity of diabetic nephropathy. *Metabolism: Clinical and Experimental*.

[31] Tisato V., Rimondi E., Brombo G. (2016). Serum soluble tumor necrosis factor-related apoptosis-inducing ligand levels in older subjects with dementia and mild cognitive impairment. *Dementia and Geriatric Cognitive Disorders*.

[32] Cervellati C., Wood P. L., Romani A. (2016). Oxidative challenge in Alzheimer’s disease: state of knowledge and future needs. *Journal of Investigative Medicine : The Official Publication of The American Federation for Clinical Research*.

[33] Niessner A., Hohensinner P. J., Rychli K. (2009). Prognostic value of apoptosis markers in advanced heart failure patients. *European Heart Journal*.

[34] Secchiero P., Corallini F., Ceconi C. (2009). Potential prognostic significance of decreased serum levels of TRAIL after acute myocardial infarction. *PLoS One*.

