# Supplementary material for: Low Circulating TRAIL Levels Are Associated with Increase of Resistin and Lipocalin-2/ngal Adipokines in Postmenopausal Women
Source: Mediators Inflamm. 2017 Sep 5;2017:5356020. doi: 10.1155/2017/5356020 (PMC5605790; doi:10.1155/2017/5356020)
Supplement: Supplementary file 1 — Supplementary Table 1. Correlation coefficient (r) between TRAIL and Adipokines. [file 5356020.f1.docx]

**Supplementary Table 1. Correlation coefficient (r) between TRAIL and Adipokines**

| **Adipokines** | **r** |
| --- | --- |
| NGF | 0.086* |
| IL-8 | 0.111* |
| TNFα | 0.135* |
| IL-6 | 0.043* |
| MCP-1 | 0.178^#^ |
| Resistin | -0.09^#^ |
| HGF | 0.041* |
| Leptin | 0.082^#^ |
| PAI-1 total | -0.026^#^ |
| Adipsin | 0.068^#^ |
| Adiponectin | 0.039^#^ |
| Lipocalin-2/ngal | -0.087^#^ |

# Pearson’s coefficient; * Spearman’s coefficient
